# 

**Published:** 1963-01

**Authors:** 


BRISTOL MEDICO-CHIRURGICAL JOURNAL

*(Incorporating the Medical Journal of the South-West)*

Established 1883

VOL. 78 (i) January 1963 NO. 287

### Contents

EDITORIAL . . . . . . . . . . 1

UNORTHODOXY IN MEDICINE AND RELIGION *A. M. Maclachlan* 2

CANCER IN AFRICA . . . *J. N. P. Davies, M.D. (Bristol)* 12

DIRECT MEASUREMENT OF NOISE LEVELS IN A LARGE HOSPITAL . . . *A. P. Cole and P. H. Dimambro* 18

"TWO CAUSES FOR MACROCYTIC ANAEMIA IN ONE PATIENT" . . . . *Clinico-Pathological Conference* 22

REPORTS FROM SOCIETIES . . . . . . . 30

NEWS FROM SOCIETIES . . . . . . . 31

*PUBLISHED QUARTERLY*

*ANNUAL SUBSCRIPTION* 16/- *POST FREE*

INSTITUTION APR 8 1963

PUBLISHED UNDER THE AUSPICES OF THE
BRISTOL MEDICO-CHIRURGICAL SOCIETY

### *Editorial Committee*

Dr. N. J. Brown, Southmead Hospital, Bristol. *Joint Editor.*

Dr. A. B. Raper, Department of Pathology, Bristol Royal Infirmary. *Joint Editor.*

Dr. J. Macrae, Ham Green Hospital.

Dr. R. C. Wofinden, Central Clinic, Bristol.

Mr. A. E. S. Roberts, Medical Library, University of Bristol. *Secretary.*

### *Local Correspondents*

*Bath* . . Mr. A. D. Bateman, 3 The Circus, Bath.

*Exeter* . . Dr. L. N. Jackson, Mount Jocelyn, Crediton, Devon.

*Gloucester* . Dr. R. F. Jarrett, 9 College Green, Gloucester.

*Plymouth* . Dr. C. R. Croft, Lexden, Hartley Av., Plymouth.

*Taunton* . Dr. M. McAlley, 1 The Crescent, Taunton.

*Torquay* . Dr. A. Robinson Thomas, 20 Devon Square, Newton Abbot, Devon.

*Truro* . . Dr. F. D. M. Hocking, St. Andrews, Perranporth, Cornwall.

*Yeovil* . . Mr. J. A. Vere Nicoll, Knapp House, Yeovil, Somerset.

### NOTICE TO CONTRIBUTORS

Original articles are invited, provided they have not been published elsewhere. Notes of forthcoming events, meetings held, degrees conferred, staff appointments, and other local news are welcomed. The editorial committee reserves the right to make literary corrections.

Illustrations should always be supplied ready for reproduction. All re-touchings or other operations required will be charged to the author. Line drawings should be drawn in Indian ink. We regret that we must ask authors to pay for making blocks, if this is necessary.

J. W. ARROWSMITH LTD., WINTERSTOKE ROAD,
BRISTOL.

### *Reports from Societies*

### BRISTOL MEDICO-CHIRURGICAL SOCIETY

The Annual General Meeting was held on 10th October at the Royal Fort. Dr. N. J. Brown showed pathological specimens and Dr. G. R. Airth demonstrated skiagrams, including a collection of remarkable films shown as a tribute to the work of the late Dr. J. V. Sparks. Mr. J. Angell James resigned the chair to his successor Dr. A. M. MacLachlan whose presidential address appears elsewhere in this issue.

Seven Honorary Members were elected on the proposal of the President; they are Mr. A. Wilfred Adams, Mr. H. Chitty, Dr. MacDonald Critchley, Mr. W. A. Jackman, Dr. H. J. Orr-Ewing, Mr. Norman Tanner and Mr. D. G. C. Tasker.

After election of the Society's officers, of whom the President Elect is to be Mr. R. V. Cooke, reports were given by the Hon. Secretary, the Hon. Treasurer and the Hon. Editor of the *Journal*. The latter announced that it would not be possible to produce the full number of issues of the *Journal* this year. This was not a new problem, it had happened many times before. Whereas, however, in the past non-appearance of the *Journal* had usually been due to financial difficulties, the reason now was a different one and arose from a lack of material for publication. It was possible that members had hesitated to offer articles because of a mistaken impression that the *Journal* had more material than it could afford to publish. This was far from being the case and any contributions from members would always be considered for publication. If sufficient material was forthcoming there was every reason to hope that all four quarterly numbers could be produced in the coming year.

### SOUTH-WEST SURGEONS

A Meeting of the Surgical Club of South-West England was held in Bristol on the 9th and 10th November 1962. On Friday the 9th, the members of the Club spent the day at the Bristol Royal Infirmary. Operations were performed by Professor R. Milnes Walker, Mr. R. G. Paul, Mr. R. E. Horton. Mr. R. H. Belsey gave a very interesting demonstration of technical matters and apparatus used in heart surgery. After this, papers were read and these covered a wide range of subjects including the functions of the pyloric antrum, pancreatitis, aortic aneurysms and the Bladder Tumour Registry. In the afternoon clinical cases were shown and this was followed by a discussion. Much of this was devoted to an account of some interesting cases of biliary surgery by Mr. R. V. Cooke.

On Saturday morning the Club were entertained by Dr. J. Macrae at Ham Green Hospital in the Respiratory Unit. A case of renal failure being dialyzed on an artificial kidney was shown. The results of the treatment of seventeen cases of renal failure were described and the practical experience gained was discussed. Dr. Macrae read a paper entitled "Fifty-four Cases of Tetanus". These patients had all come from the South-West of England and brought home to everyone the fact that this disease was still with us. In spite of the excellent treatment given to these patients, some of them had died because of the toxicity of the organism. Prophylaxis was discussed, and active immunization against tetanus was advocated. It was pointed out that there was no such thing as a typical tetanus wound and that the repeated administration of anti-tetanic serum was both useless and harmful, in that the patient developed a resistance to the serum. In conclusion, there was a demonstration of the monitor systems used during artificial respiration.

### WESSEX CLINICAL PATHOLOGISTS

A meeting of the Wessex Branch of the Association of Clinical Pathologists was held at Barnstaple on Saturday 11th November 1962. Four papers were read, and demonstrations were shown.

Dr. H. Miller and Mr. J. A. Durant (Portsmouth) had compared three methods of testing for pregnancy; the standard Hogben toad method, and two commercially produced immunological tests (*a*) that of Ortho Pharmaceuticals, which employs a latex particle suspension, and (*b*) the Prepuerin test (Burroughs Wellcome) which uses sheep red cells. These two tests are designed to detect human chorionic gonadotrophin in the urine. Out of 234 women tested it was possible to ascertain later whether pregnancy had been present or not in 159. The results in these 159 subjects were as follows:

| Test | Number tested | No. correct | False +ves | False −ves |
|---|---|---|---|---|
| Hogben .. .. .. | 159 | 148 | 0 | 11 |
| "Ortho" .. .. .. | 159 | 144 | 5 | 10 |
| "Prepuerin" .. .. | 76 | 70 | 2 | 4 |

False negative results occurred about equally with all three tests. False positives occurred in the two immunological tests, but not in the biological toad test. Since reliance on a positive test has important consequences, the authors proposed to continue to rely upon the Hogben test.

### *News from Societies*

### EXAMINATION RESULTS

Final M.B., Ch.B. (Bristol), December 1962.

| | |
|---|---|
| Abbott, A. P. | Moreton, T. J. |
| Butler, J. S. | Jones, J. J. |
| Ezeji-Okeye, O. | Drion, M. |
| Halwood, C. C. | Yap, C. H. |

Professor R. Milnes Walker delivered the Skinner Lecture at the Royal College of Surgeons of London on 15th November 1962 on "Cancer treatment then and now".

Professor R. Milnes Walker was elected to Honorary Fellowship of the American College of Surgeons on 18th October 1962.

*Printed* [illegible]

J. W. Arrowsmith Ltd., Bris[illegible]

